# An Ordinary State-Based Peridynamic Model of Unidirectional Carbon Fiber Reinforced Polymer Material in the Cutting Process

**DOI:** 10.3390/polym15010064

**Published:** 2022-12-23

**Authors:** Jiaqi Qi, Cheng Li, Ying Tie, Yanping Zheng, Zhen Cui, Yuechen Duan

**Affiliations:** School of Mechanical and Power Engineering, Zhengzhou University, No. 100 Science Avenue, Zhengzhou 450001, China

**Keywords:** peridynamics, anisotropic material, failure, orthogonal cutting

## Abstract

Due to the complexity of the composite structure, analyzing the material failure process of carbon fiber reinforced polymers (CFRP) is fairly difficult, particularly for the machining process. Peridynamic theory, a new branch of solid mechanics, is a useful tool for dealing with discontinuities. This study presents an ordinary state-based peridynamic (OSB-PD) model for unidirectional CFRP material in the cutting process. In this model, angle tolerance is used to overcome the fiber angle limitation in a classical OSB-PD laminate method, and the short-range force approach is utilized to simulate the contact of the cutting tool and workpiece. The effectiveness of the supplied models is validated by tension and cutting tests. Finally, it can be indicated that the OSB-PD model is capable of predicting machined surface damage and cutting force, based on the comparison of simulation and experimental data.

## 1. Introduction

Load-bearing components made of laminated composites are widely employed in modern aviation and automobile systems. This is due to the fact that laminated composites have an excellent strength-to-weight ratio, high specific stiffness, and superior fatigue resistance [1]. In engineering applications, the machining of CFRP material is a necessary step that must be taken in order to fulfill the requirements of assembly. However, due to the fact that CFRP exhibits anisotropy, quasi-brittleness, and multiple-interface properties, the machining process might be significantly complicated by it [2]. As computer technology has evolved, numerical simulations have become more common. These simulations help researchers to better understand the constitutive behavior and failure processes of many types of materials and structures, which has contributed to their popularity. The finite element method (FEM) is now extensively utilized to quantitatively simulate composite damage characteristics [3,4,5]. However, the finite element method has its limitations when it comes to dealing with issues involving discontinuities. Because its fundamental motion equation is constructed from a partial differential form of the displacement fields, these equations do not make sense on the tip of a crack. As a direct result of this, several strategies have been presented as potential solutions to this issue, including external fracture growth criteria, re-meshing technology, and many more [6].

The strong nonlocal formulation of continuum mechanics known as peridynamics (PD) was first conceived of and developed by Silling of Sandia National Lab [7]. In this theory, the partial derivative equations of classical continuum theories (CCTS) are recast as integral–differential equations. These types of equations are well-suited for the task of handling issues involving discontinuities [8,9]. Because PD provides so many advantages in the analysis of material failure, numerical simulations based on the PD theory in the study of isotropic solids [10,11,12] and composites [13,14,15,16] are gaining popularity. On the basis of this theory, scholars have conducted research into the corrosion damage of metal materials [12] and the damage of composite laminates under quasi-static [13,14,15] and fatigue loading [16].

Currently, utilization of PD theory to explore the mechanical properties and damage propagation of composite structures has increased. In [17], the peridynamic simulation of quasi-static tensile failure and dynamic impact damage of laminated composite was investigated for the first time. Kilic et al. [13] created a PD model with fiber and matrix at separated material points and predicted matrix cracking in laminated composites, taking into account the nonuniform unique characteristics of the fiber and matrix. The numerical results were consistent with experimental data. Ghajari et al. [18] developed a continuous model with spherical harmonic expansion to approximate the bond stiffness function for orthotropic media, taking into account that stiffness continually changes from fiber orientation to the perpendicular direction. Hu and Yu [19] introduced a PD model of laminated material that can capture various material couplings in transverse shear deformation. The numerical results agreed well with the test results. A peridynamic method for simulating composite laminates based on PD differential operator was recently proposed by Madenci et al. [20]. It not only removes the restrictions placed on the fiber direction and the material properties, but it also enables the evaluation of stress fields, which were initially utilized in classical continuum theories. Tian et al. [21] introduced a continuum-kinematics-inspired peridynamic (CPD) model of anisotropic material. The micro moduli, critical stretch, and critical micro potential energy in the CPD model are continuous functions, which is achieved by eight-order double Fourier series. In addition to this, there are also a few novel uses in the process of metal machining [22,23]. Therefore, it can be inferred that PD theory is suitable for analyzing the breakage of various materials.

As noted above, previous research mainly focused on the analysis of tensile damage and impact fracture of composite materials. There are few PD models that have been established for the CFRP materials cutting process. Although Shang et al. have built a bond-based peridynamic (BB-PD) model [24] and a non-ordinary state-based peridynamic (NOSB-PD) model [25] to analyze the unidirectional carbon fiber reinforced polymer (UD-CFRP) material cutting process, the former is born with Poison’s ratio limitation and the latter with zeros energy mode restriction. The ordinary state-based peridynamic laminated theory (OSB-PDLT) introduced by Oterkus [26] is a classical model for laminated composites. It does not suffer from the drawbacks of the aforementioned models.

To the authors’ knowledge, there is still no PD modeling built for the CFRP materials cutting process based on the OSB-PDLT approach. In addition, Shang et al. [24] only investigated the fiber orientation of $0^{\circ }$, ±$45^{\circ }$, and $90^{\circ }$, which was a result of the limitation of the specific material point distribution. In this study, an improved OSB-PD CFRP model is proposed to remove the fiber orientation restriction so that the model can be applied to general fiber orientations.

A precise prediction of the cutting process is a meaningful work in the machining engineering of composite materials. Therefore, in this paper, an application of OSB-PDLT theory is presented in the form of an analysis of the cutting process of CFRP materials. The remaining parts of the paper are organized as follows: In Section 2, the modeling of the machining of UD-CFRP materials is presented. In Section 3, The numerical findings and the experimental results are compared with one another [25], and the results are discussed in detail. Section 4 provides a synopsis of the main findings.

## 2. Modeling of Machining of UD-CFRP Materials

### 2.1. A Brief Review of the OSB-PD Model

**Figure 1:**
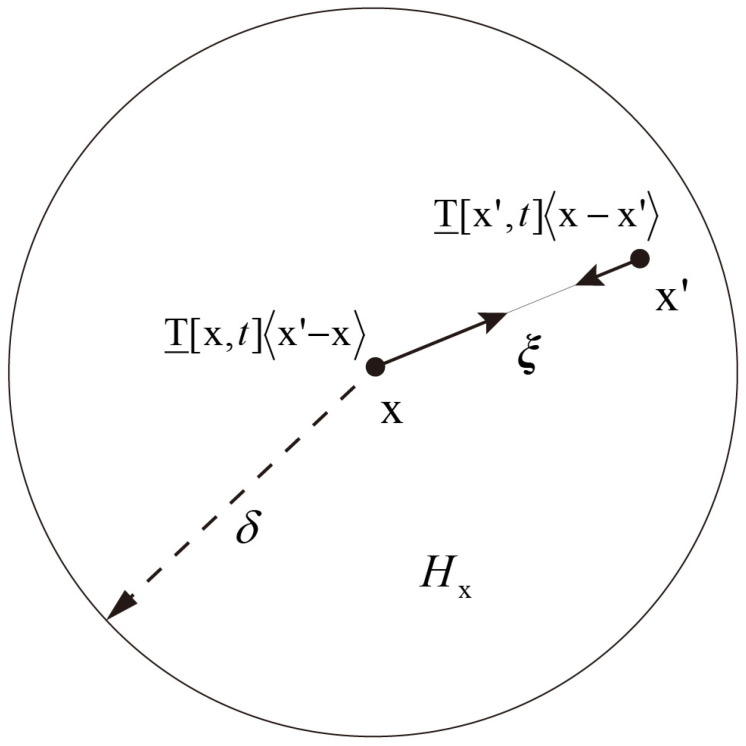
OSB-PD model.

Integral–differential equations are used in peridynamic theory, eliminating the partial derivatives of the deformation in terms of spatial coordinates [7,8,9,26]. The horizon, δ, defines the region in which a particle can have an effect on other points, as shown in Figure 1. The PD equilibrium equations continue to remain applicable even after the interactions between particles have ceased to exist, despite the fact that this may cause a crack to initiate and propagate along surfaces that have formed cracks.

In the generalized OSB-PD model, the main equation of the material point x can be written as [26]:

(1) $$\rho \left(x\right)\ddot{u}\left(x,t\right)=\int_{H_{x}}^{}\left\{\underline{T}\left[x,t\right]\langle x^{\prime }-x\rangle -\underline{T}\left[x^{\prime },t\right]\langle x-x^{\prime }\rangle \right\}dV_{x^{\prime }}+b\left(x,t\right),$$

where ρ represents density, $\ddot{u}$ is the acceleration. $x^{\prime }$ is the material point within the neighborhood, $H_{x}$, and b(x,t) is the body force density. As shown in Figure 1, ξ represents the bond vector; $\underline{T}$ represents the force vector state.

### 2.2. PD Laminated Composite Theory

**Figure 2:**
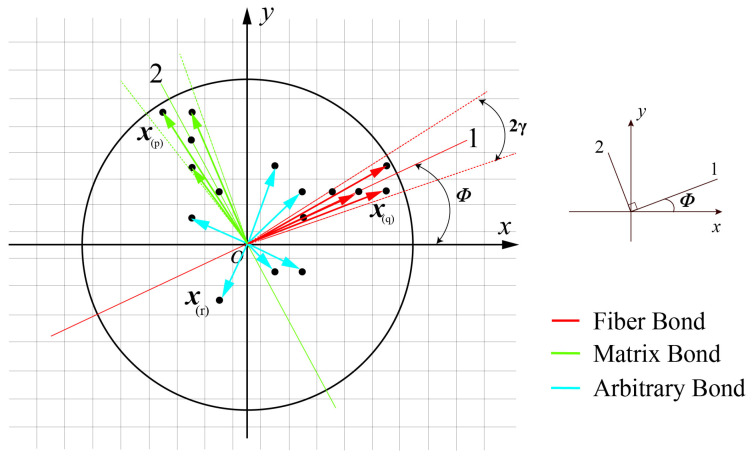
The PD model diagrammatic drawing for a single layer.

Each FRP lamina can be idealized as a 2D structure based on the directionality of the contacts between the PD material points. After grid discretization in the present PD model for a lamina, the particles and 1- and 2-principal material axes should be determined, as illustrated in Figure 2. Fiber bonds, matrix bonds, and arbitrary bonds are the three categories used to describe the interactions between any two particles. In [26], because fiber bonds may only be assigned to material sites along the precise orientation of φ, the discretization grid may not line up with the anticipated 1- and 2-principle directions [27]. This may result in the fiber angle in the PD model being limited to a specific value, such as $0^{\circ }$, ±$45^{\circ }$, and $90^{\circ }$. Referring to [28,29], γ for fiber and matrix bonds are both set as ±$5^{\circ }$ in this research; $x_{\left(q\right)}$ denotes material points along the 1-principle axis that interact with the target material point $x_{\left(k\right)}$, as shown in Figure 2; $x_{\left(p\right)}$ denotes material points that interact with $x_{\left(k\right)}$ along the 2-principle axis; $x_{\left(r\right)}$ denotes the points within the horizon in any direction [30].

According to Madenci’s work [26], the equation of motion for $x_{\left(k\right)}$ in a lamina can be expressed as

(2) $$\begin{array}{c} \rho_{\left(k\right)}{\ddot{u}}_{\left(k\right)}=\sum_{j=1}\left[t_{\left(k)(j\right)}\left(u_{\left(j\right)}-u_{\left(k\right)},x_{\left(j\right)}-x_{\left(k\right)},t\right)-t_{\left(j)(k\right)}\left(u_{\left(k\right)}-u_{\left(j\right)},x_{\left(k\right)}-x_{\left(j\right)},t\right)\right]V_{\left(k\right)}+b_{\left(k\right)} \end{array}$$

where the particle $x_{\left(k\right)}$ has an incremental volume, $V_{\left(k\right)}$; t designates time [30]; $x_{\left(j\right)}$ represents a particle being a family member of node $x_{\left(k\right)}$; $t_{\left(k)(j\right)}$ is used to represent the PD force density vector; and $y_{\left(k\right)}$ represents the new relative position vector of the two particles. The force density vectors are derived by Madenci and Oterkus [26] in the form

(3) $$t_{\left(k)(j\right)}\left(u_{\left(j\right)}-u_{\left(k\right)},x_{\left(j\right)}-x_{\left(k\right)},t\right)=2\delta \left(ad\frac{\Lambda_{\left(k)(j\right)}}{\left|x_{\left(j\right)}-x_{\left(k\right)}\right|}\theta_{\left(k\right)}+\left(\mu_{F}b_{F}+b_{FT}+\mu_{T}b_{T}\right)s_{\left(k)(j\right)}\right)\frac{y_{\left(j\right)}-y_{\left(k\right)}}{\left|y_{\left(j\right)}-y_{\left(k\right)}\right|}$$

(4) $$t_{\left(j)(k\right)}\left(u_{\left(k\right)}-u_{\left(j\right)},x_{\left(k\right)}-x_{\left(j\right)},t\right)=2\delta \left(ad\frac{\Lambda_{\left(j)(k\right)}}{\left|x_{\left(k\right)}-x_{\left(j\right)}\right|}\theta_{\left(j\right)}+\left(\mu_{F}b_{F}+b_{FT}+\mu_{T}b_{T}\right)s_{\left(j)(k\right)}\right)\frac{y_{\left(k\right)}-y_{\left(j\right)}}{\left|y_{\left(k\right)}-y_{\left(j\right)}\right|}$$

with

(5) $$s_{\left(k)(j\right)}=\frac{\left|y_{\left(j\right)}-y_{\left(k\right)}\right|-\left|x_{\left(j\right)}-x_{\left(k\right)}\right|}{\left|x_{\left(j\right)}-x_{\left(k\right)}\right|}$$

and

(6) $$\mu_{F}=\left\{\begin{array}{cc} 1, & \;\mathrm{fiber}\;\mathrm{bond}\;\\ 0, & \;\mathrm{otherwise}\; \end{array}\right.$$

and

(7) $$\mu_{T}=\left\{\begin{array}{cc} 1, & \;\mathrm{matrix}\;\mathrm{bond}\;\\ 0, & \;\mathrm{otherwise}\; \end{array}\right.$$

The parameter $\theta_{\left(k\right)}$ is defined as

(8) $$\theta_{\left(k\right)}=d\sum_{j=1}\frac{\delta }{\left|x_{\left(j\right)}-x_{\left(k\right)}\right|}\left(\left|y_{\left(j\right)}-y_{\left(k\right)}\right|-\left|x_{\left(j\right)}-x_{\left(k\right)}\right|\right)\Lambda_{\left(k)(j\right)}V_{\left(j\right)}$$

The peridynamic auxiliary parameter $\Lambda_{\left(k)(j\right)}$ is defined as

(9) $$\Lambda_{\left(k)(j\right)}=\frac{y_{\left(j\right)}-y_{\left(k\right)}}{\left|y_{\left(j\right)}-y_{\left(k\right)}\right|}\cdot \frac{x_{\left(j\right)}-x_{\left(k\right)}}{\left|x_{\left(j\right)}-x_{\left(k\right)}\right|}$$

The peridynamic material parameters in Equation (3) are defined as [26]:

(10) $$a=\frac{1}{2}\left(Q_{12}-Q_{66}\right)$$

(11) $$d=\frac{2}{\pi h\delta^{3}}$$

(12) $$b_{F}=\frac{\left(Q_{11}-Q_{12}-2Q_{66}\right)}{2\delta \left(\sum_{j=1}^{J_{F}}\left|x_{\left(j\right)}-x_{\left(k\right)}\right|V_{\left(j\right)}\right)}$$

(13) $$b_{T}=\frac{\left(Q_{22}-Q_{12}-2Q_{66}\right)}{2\delta \left(\sum_{j=1}^{J_{T}}\left|x_{\left(j\right)}-x_{\left(k\right)}\right|V_{\left(j\right)}\right)}$$

(14) $$b_{FT}=\frac{6Q_{66}}{\pi h\delta^{4}}$$

where

(15) $$Q_{11}=\frac{E_{11}}{1-\nu_{12}\nu_{21}},Q_{12}=\frac{\nu_{12}E_{22}}{1-\nu_{12}\nu_{21}},Q_{22}=\frac{E_{22}}{1-\nu_{12}\nu_{21}},Q_{66}=G_{12},Q_{66}=G_{12}.$$

The peridynamic material parameters $b_{F}$, $b_{T}$, and $b_{\mathrm{FT}}$ are related to the deformation of particles in the fiber direction, transverse direction, and remaining arbitrary directions, respectively; h represents the thickness of the plate. In Equation (15), $E_{11}$, $E_{22}$, $G_{12}$, $\nu_{12}$, and $\nu_{21}$ represent the elastic modulus in fiber direction, the elastic modulus in transverse direction, major shear modulus, major Poisson’s ratio, and minor Poisson’s ratio, respectively.

### 2.3. Failure Criterion

PD failure is generally emulated by breaking the bonds irreversibly. In the event that the changed value of a bond exceeds the critical value in a brittle fracture condition, the bond will be broken. Due to the complex construction, determining the critical stretch value for laminated material is usually difficult. In recent investigations, there have been many approaches to determine the failure of PD bonds [31,32,33,34,35,36,37]. In this paper, the failure prediction is based on a relatively convenient approach, which is calculated by the mechanical strengths of a lamina [33,34,35]. The values of the critical stretch for in-plane fiber bonds, in-plane matrix bonds, and remaining arbitrary bonds [35] can be calculated as

(16) $$\left\{\begin{array}{cc} s_{0}^{\mathrm{ft}}=\frac{X^{T}}{E_{11}}, & s\geq 0\;(\mathrm{fiber}\;\mathrm{bonds})\;\\ s_{0}^{\mathrm{fc}\;}=\frac{X^{C}}{E_{11}}, & s<0\;(\mathrm{fiber}\;\mathrm{bonds})\;\\ s_{0}^{\mathrm{mt}}=\frac{Y^{T}}{E_{22}}, & s\geq 0\;(\mathrm{matrix}\;\mathrm{bonds}\;\mathrm{and}\;\mathrm{abitrary}\;\mathrm{bonds})\;\\ s_{0}^{\mathrm{mc}}=\frac{Y^{C}}{E_{22}}, & s<0\;(\mathrm{matrix}\;\mathrm{bonds}\;\mathrm{and}\;\mathrm{abitrary}\;\mathrm{bonds})\; \end{array}\right.$$

where the $X^{T}$, $X^{C}$, $Y^{T}$, and $Y^{C}$ are strengths of composite materials. The damage level at a particle is represented by [8,26]

(17) $$\varphi \left(x,t\right)=1-\frac{\int_{H_{x}}\mu \left(x^{\prime }-x,t\right)dV_{x^{\prime }}}{\int_{H_{x}}dV^{\prime }}.$$

### 2.4. Contact Algorithm

**Figure 3:**
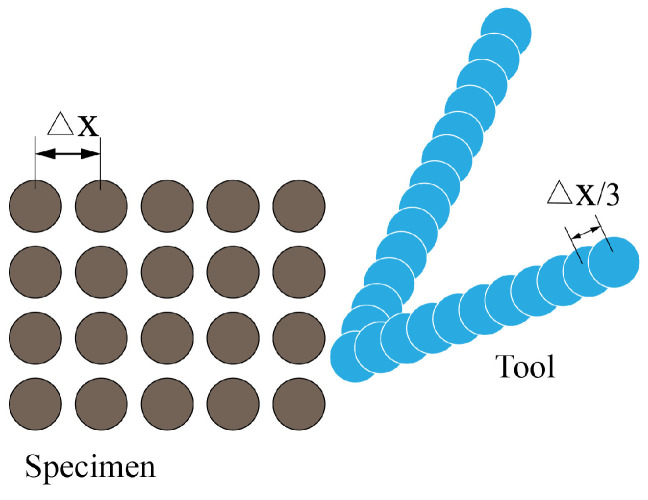
Diagrammatic drawing of the PD cutting model.

**Figure 4:**
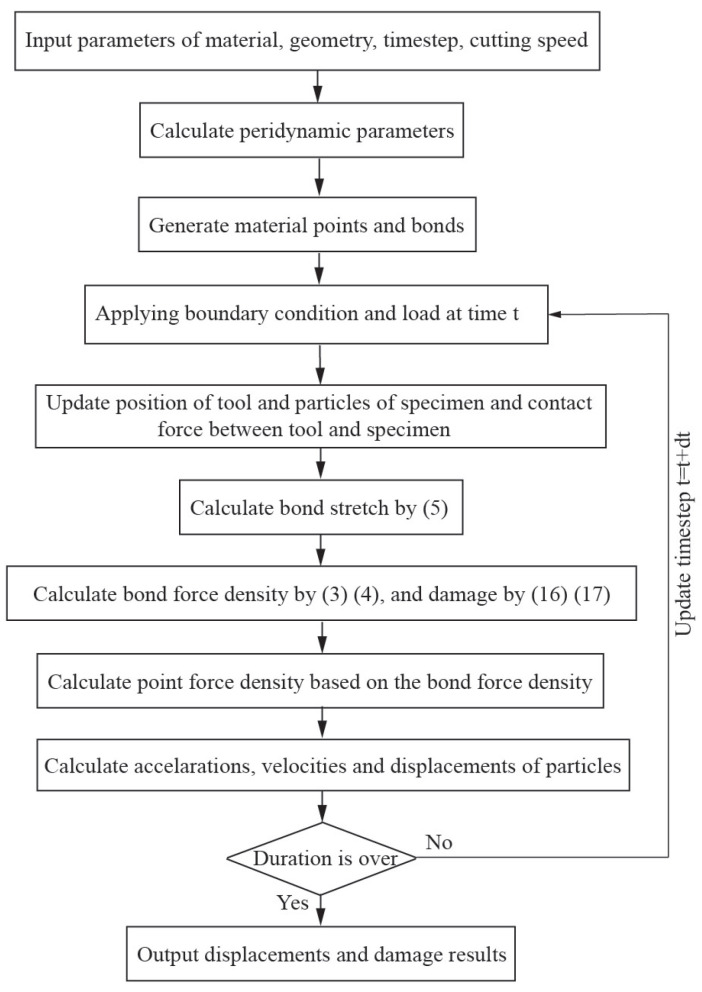
Flow chart for the laminated composites cutting PD program.

An appropriate and accurate contact method is very crucial in the modeling of the cutting process. To prevent the interpenetration between bodies in the PD analysis, Silling and Askari [8] implemented the short-range force approach to solve such contact problems. The expression for the short-range force between material points is:

(18) $$f_{\mathrm{sh}}\left(y_{\left(j\right)}-y_{\left(k\right)}\right)=\frac{y_{\left(j\right)}-y_{\left(k\right)}}{\left|y_{\left(j\right)}-y_{\left(k\right)}\right|}\min\left\{0,c_{\mathrm{sh}}\left[\frac{\left|y_{\left(j\right)}-y_{\left(k\right)}\right|}{2r_{\mathrm{sh}}}-1\right]\right\}$$

where the short-range force constant, $c_{\mathrm{sh}}$, can be decided by the BB-PD material parameter *c* [26]; $r_{\mathrm{sh}}$ is the critical distance, which is chosen as ▵x in this paper, where the parameter ▵x is the distance between two adjacent particles along the principal axis in the in-plane workpiece. This method enables the avoidance of material specimen points going through the cutting instrument, as shown in Figure 3. Additionally, Figure 4 depicts the coding flow chart used in this study for laminated composites throughout the cutting process.

### 2.5. Numerical Implementation

Similar to Silling [8], a uniform grid size ▵x is used; δ is the horizon size, δ=m▵x. There are introduced fictitious material layers to the constraint border. In this research, we use the force–density-based techniques for correcting the surface effect of the PD model [26]. To maximize the effectiveness of computing, a strategy known as adaptive dynamic relaxation (ADR) [10] is used.

## 3. Results and Discussion

### 3.1. Tension Simulation

**Figure 5:**
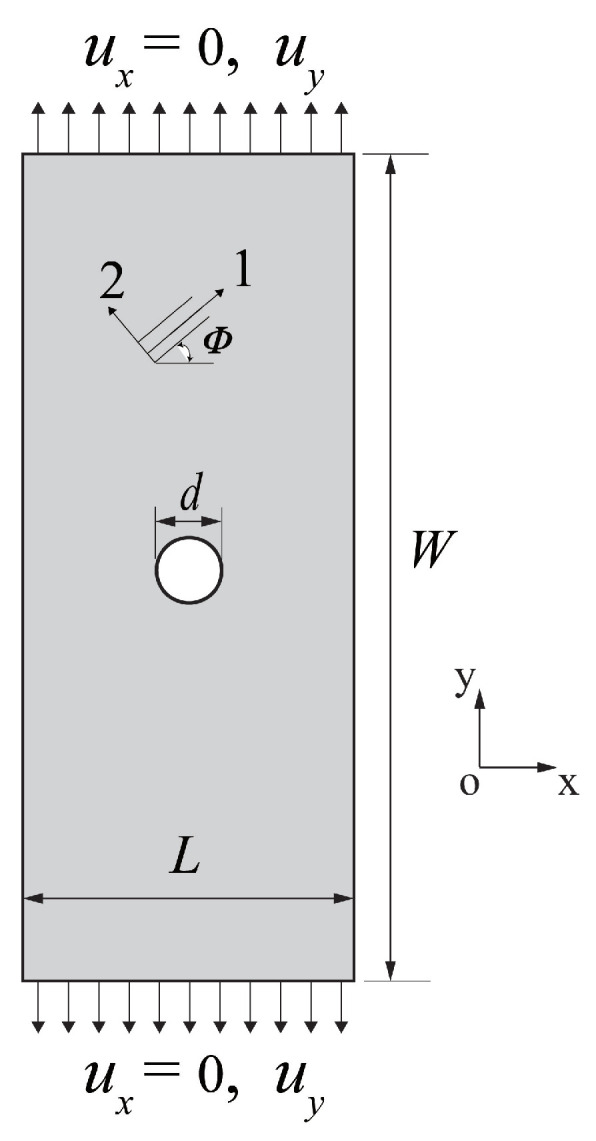
Sketch map for a lamina with a hole under tensile loading.

A lamina with a central hole is utilized to demonstrate the effectiveness of the OSB-PDLT modeling. The material of the specimen is T300 carbon fiber reinforced polymer composite, and its material properties are illustrated in Table 1 [25]. As illustrated in Figure 5, the lamina has dimensions of 50 mm in length, 100 mm in breadth, and 1 mm in thickness. The diameter *d* of the hole is 10 mm. The top and lower borders of the material plate are subjected to the real experiment’s force load and boundary conditions. In this tension test, a uniform grid 150×300 was chosen to be used in the discretization process. The radius of the horizon is set as 3▵x to improve the calculation speed. The ADR approach was used in unity time increments. The stable mass density take the value of $2.127\times 10^{10}$ kg/m${}^{3}$. There are a total of 2000 time steps in the process.

**Figure 6:**
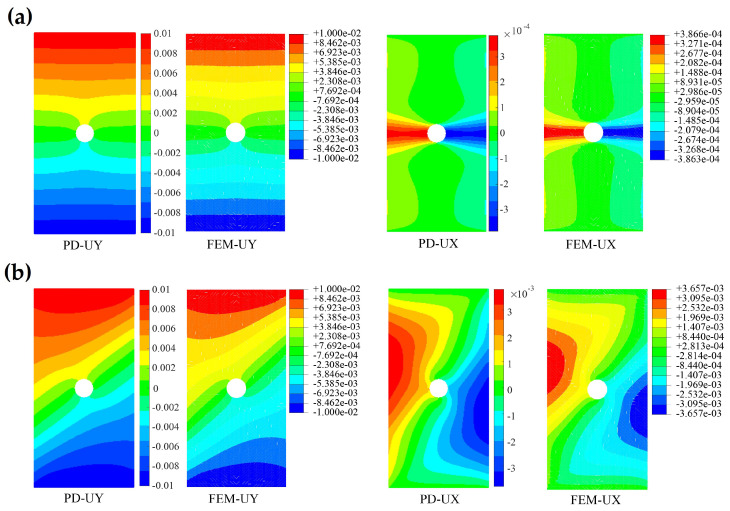
Displacement fields of laminas with different fiber directions. (**a**) Comparison of the displacement fields of $0^{\circ }$ open-hole lamina under tensile loading (mm). (**b**) Comparison of the displacement fields of $45^{\circ }$ open-hole lamina under tensile loading (mm).

**Figure 7:**
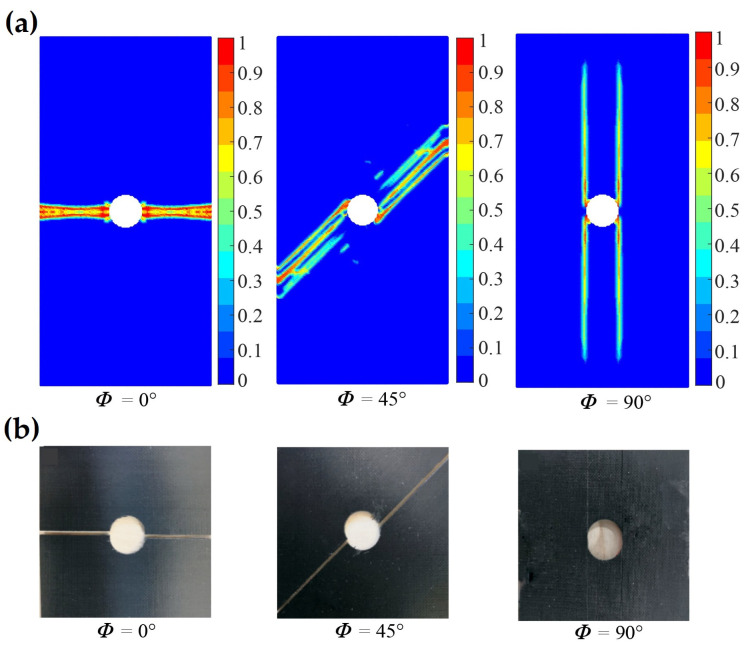
Damage of laminas of different fiber directions. (**a**) Simulation results for different fiber directions. (**b**) Experimental results for different fiber direction [25].

For the uniaxial tensile test of a lamina, the fiber directions of $0^{\circ }$ and $45^{\circ }$ are selected. In the stage of elastic deformation, the displacement fields calculated by PD and FEM of the laminas are shown in Figure 6. It can be found that the $u_{x}$ and $u_{y}$ displacement fields obtained by the OSB-PDLT model are in good agreement with the FEM results. In addition, in order to demonstrate that the OSB-PDLT model is capable of accurately predicting failure, the results of testing the model with three distinct fiber orientations are shown in Figure 7. As shown in Figure 7, the main damage mode is matrix cracking along the fiber direction, and the numerical results of PD correlate very well with experimental observation [25]. The validity of the lamina model is fully proved by the above simulation results.

### 3.2. Cutting Process for CFRP Lamina

#### 3.2.1. Experimental and Simulation Setup

**Figure 8:**
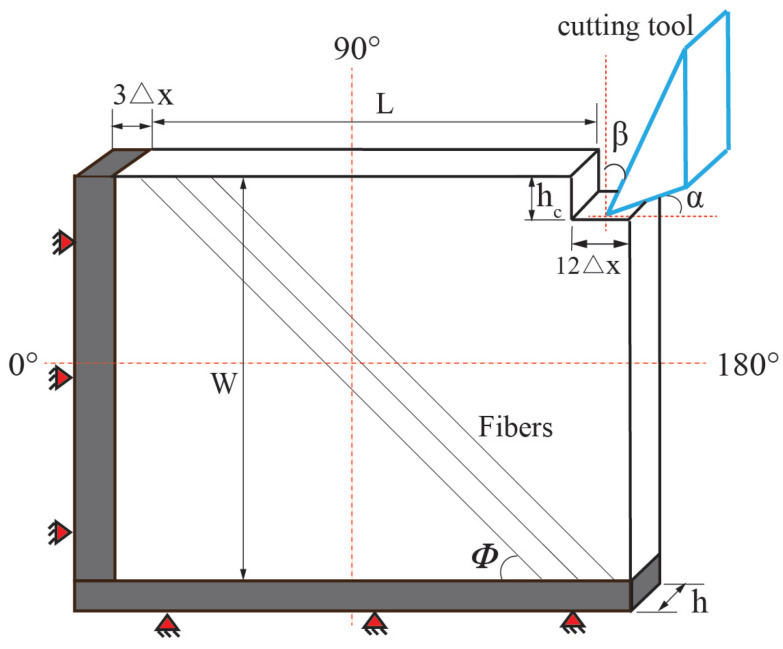
Schematic of simulation setup for CFRP orthogonal cutting.

The experimental results of this study are based on the research provided in [25]. The illustration as well as the explanation of the results may be found in [25], and the purpose of this part is to only provide a concise introduction to the substance of the exam. The length of the cutting edge is 7 mm, which is more than the thickness of the specimen. To guarantee that there was no rotational behavior throughout the testing, the tool spindle was locked. Figure 8 depicts a schematic of CFRP orthogonal cutting simulation settings. The clearance angle α is $20^{\circ }$. Rake angle β of the cutting tool is $25^{\circ }$. In this section, the selected fiber angles were $0^{\circ }$, $45^{\circ }$, $90^{\circ }$, and $135^{\circ }$. The cutting depth is fixed at $h_{c}$ = 0.2 mm. The cutting velocity is 0.6 m/min, and the cooling is done in a dry environment. This lamina’s dimensions are L=8 mm and W=8 mm, with h=1 mm being its thickness. The specimen we used here is the unidirectional T300 carbon fiber/epoxy composite. Its material parameters are shown in Table 1 [25].

In this section, the specimen is discretized as a uniform grid 300×300. In addition, some missing material points in the upper right corner are ignored. The boundary conditions, cutting speed, and cutting depth in the PD simulation are similar to the cutting experiment. The cutting tool and the particles in the boundary are defined as rigid bodies, whereas the latter cannot be moved. In addition to that, the models do not take into account the friction [24,25]. There are a total of 4000 time steps in the process. As the fiber angles investigated are relatively conventional, the radius is set as 3▵x to improve the calculation efficiency. The short-range force constant $c_{sh}=0.1c$ was adopted [25].

#### 3.2.2. Cutting Process under General Fiber Orientations

**Figure 9:**
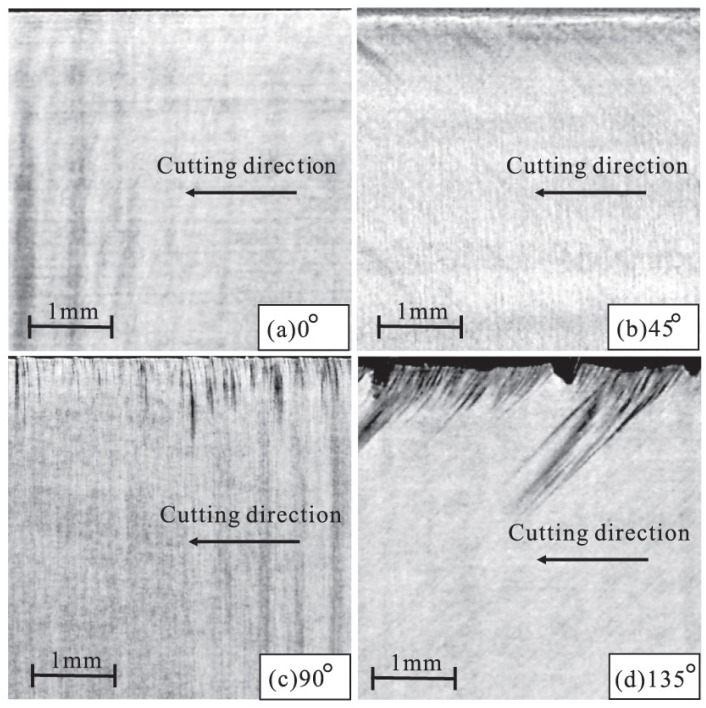
Schematic of simulation setups of CFRP orthogonal cutting [25].

**Figure 10:**
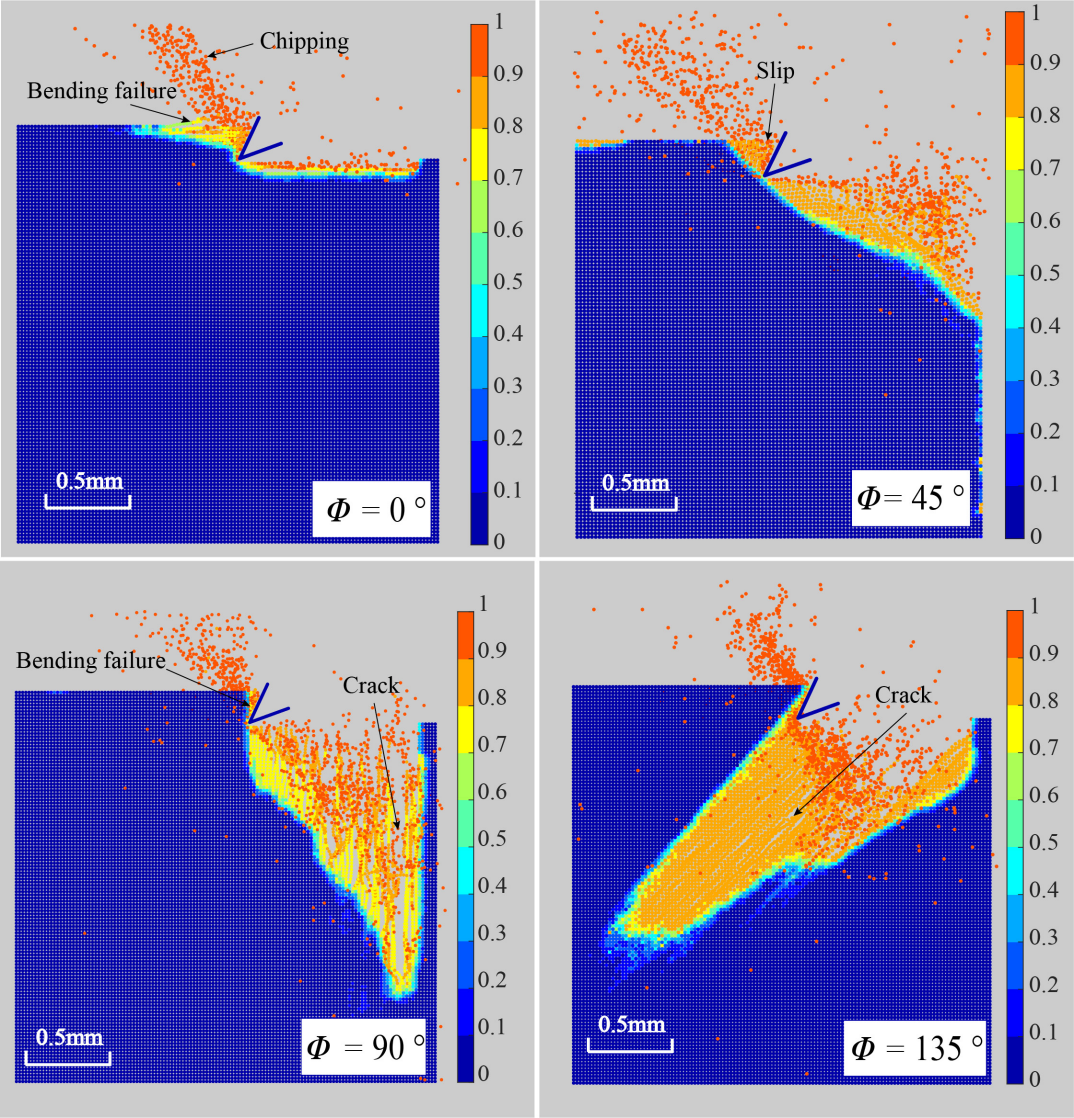
Chip formation for $\phi =0^{\circ },45^{\circ },90^{\circ },135^{\circ }$ (partial enlarged drawing).

Fiber angle ϕ has great influence on the cutting surface damage and chip formation. Figure 9 depicts the machined surface of the experimental results [25] for different fiber orientations. Figure 10 depicts the corresponding modeling results. Based on Figure 9 and Figure 10, it can be seen that when the fiber angle rises, sub-surface damage increases [25], which is consistent with the findings in [25,38]. In addition, from the numerical results, we can find that when the fiber angle is $0^{\circ }$, the machined surface is smooth and the damage depth is quite minimal. Because crack always propagates along the track that requires the least energy and the strength of the fiber is significantly greater than the strength of the matrix, the machined surface crack always grows along the fiber direction. From the simulation results of chip formation of $\phi =0^{\circ }$ and $\phi =135^{\circ }$, it was discovered that the crack propagates ahead of the tool along the fiber path. For the fiber angle of $\phi =90^{\circ }$ and $\phi =135^{\circ }$, we observe obvious diffuse damage in the matrix and splitting fracture mode from Figure 10. By comparison with the results in [25], it can also be found that the OSB-PDLT in the cutting process modeling presented in this paper can simulate the damage characteristics of laminas with different fiber angles in the cutting process, which cannot be realized by the NOSB-PD model [31].

**Figure 11:**
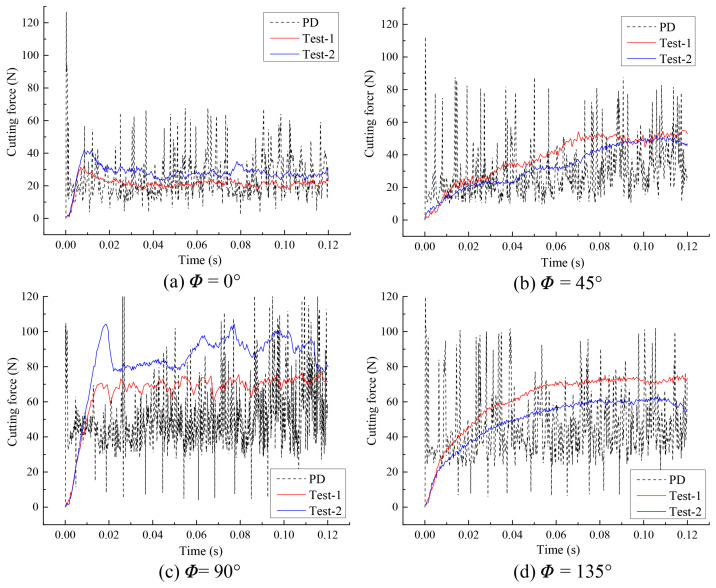
Comparison of simulation and experiment cutting force for different fiber orientations.

**Figure 12:**
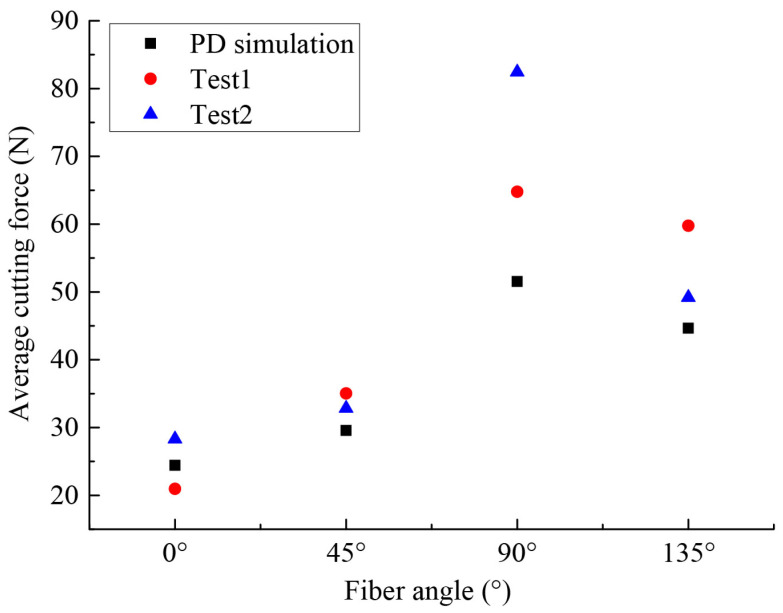
Average cutting force for different fiber angles.

To further verify the effectiveness of the OSB-PDLT in cutting process modeling, the cutting force of simulation and experiment results were also compared, as shown in Figure 11. We see that the numerical results based on this improved model closely match the results of the experimental cutting force tests [25]. However, numerical oscillations in PD results were found when evaluating the cutting force. This numerical oscillation might be related to the bond failure evaluation method, which can be effectively suppressed by considering the new bond-failure approach [39]. The average values of cutting force in numerical and experimental tests [25] are illustrated in Figure 12. We find that the cutting force is lowest when the $\phi =0^{\circ }$. The highest cutting force comes from the $\phi =90^{\circ }$. These findings agree well with the conclusion in [25].

#### 3.2.3. Cutting Process for Different Fiber Orientations

**Figure 13:**
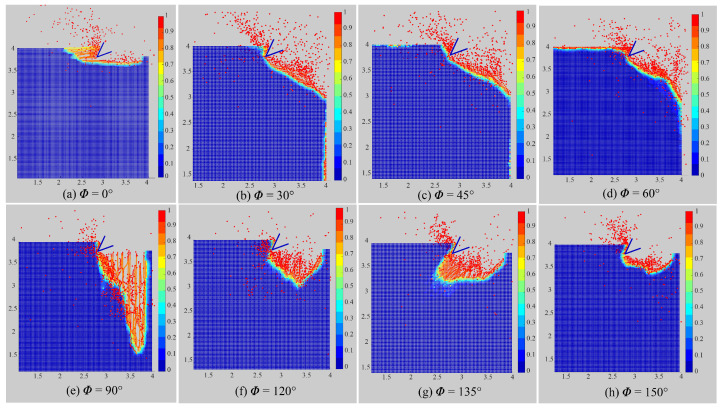
Chip formation for different fiber orientations (partial enlarged drawing).

In order to meet different engineering requirements, the cutting process will encounter plates of different fiber angles. In this section, different material orientations $\phi =0^{\circ },30^{\circ },45^{\circ },60^{\circ },90^{\circ },120^{\circ },135^{\circ }$, and $150^{\circ }$ are investigated for the cutting test. The numerical parameters all remain unchanged except the horizon radius. The horizon radius is set as 4▵x to make more material points locate in the horizon. Figure 13 shows the machined surface of numerical results for various fiber angles.

Therefore, the model in cutting process established in this study can well simulate the damage forms of laminas with different fiber angles. It can be observed that the damage depth of $0^{\circ }$ lamina is the smallest, and the damage depth of $90^{\circ }$ lamina is the largest. A second conclusion that can be drawn from Figure 13 is that chips are created when the matrix–fiber interface is sheared along the fiber orientation when the angle of incidence ranges from 0 to 90 degrees. In the cutting process, the roughness of the specimen surface may be degraded by the fiber bouncing back. It is mainly affected by the fiber angle in the case where $90^{\circ }\leq \phi \leq 150^{\circ }$. The simulation findings correlate well with the experimental results presented in [38], and it denotes that this OSB-PDLT modeling is able to give good predictions of laminas in the cutting process. Generally, it is hard for FEM to calculate such complex numerical models and get such damage patterns when faced with severe element distortion and extra failure criterion. In contrast, the PD method is inherently good at solving the fracture problems.

## 4. Conclusions

To properly simulate the failure and fracture behaviors of the machining process for CFRP, a robust numerical methodology is essential. To accomplish this objective, this work provides a composite modeling based on the OSB-PDLT approach. The interplay behavior between the cutting pattern and the specimen is modeled using the short-range force approach. The proposed modeling was tested using tensile and cutting simulations of CFRP lamina.

By comparing and analyzing the simulation and experiment results, the following are some of the inferences that can be drawn:

(1) The consistency between numerical results and experimental findings [25,38] indicates the validity of the provided model. In addition, the simulation results allow one to see the details of machined surface degradation, chip development, and fracture growth.
(2) Fiber direction has a great influence on the quality of the machining surface. In this study, the quality of the machined surface of the plate with $\phi =0^{\circ }$ is the best, whereas the plate with $\phi =90^{\circ }$ is the worst, according to the depth of cutting damage.

Therefore, we can infer that the provided method is capable of supplying the desired result in the analysis of the CFRP lamina cutting process. However, in order to get more accurate simulation results, the proposed model needs improvement. For instance, numerical oscillation might be effectively suppressed by considering some new bond-failure approaches. In addition, this PD modeling for the cutting process can be further extended for multi-orientation CFRP structure.

## Figures and Tables

**Table 1:** Mechanical properties of the CFRP [25].

Mechanical Property	Value
$E_{1}\left(\mathrm{MPa}\right)$	133,000
$E_{2}\left(\mathrm{MPa}\right)$	8000
*v*	0.33
$G_{12}\left(\mathrm{MPa}\right)$	3700
$X_{t}\left(\mathrm{MPa}\right)$	1900
$X_{c}\left(\mathrm{MPa}\right)$	1300
$Y_{t}\left(\mathrm{MPa}\right)$	41
$Y_{c}\left(\mathrm{MPa}\right)$	170
$S_{c}\left(\mathrm{MPa}\right)$	81

## Data Availability

Data presented in this study are available on request from the corresponding author.
